# 2025 International Urology Cancer Summit #IUCS25 abstracts

**DOI:** 10.1093/oncolo/oyaf248.001

**Published:** 2025-08-29

## Abstract

The third edition of the International Urology Cancer Summit (IUCS25) on September 4-5, 2025, in Portsmouth, United Kingdom, is a hybrid event combining in-person sessions with a virtual platform to maximize accessibility and engagement. The Summit brings together global leaders in urologic oncology to share the latest advances across prostate, kidney, bladder, and testicular cancers. The scientific program features keynote lectures, state-of-the-art updates, oral abstract sessions, and dedicated thematic tracks designed to foster in-depth discussion and collaboration.

IUCS is a free and independent educational initiative with no registration cost, designed to support equitable access to high-quality scientific exchange. The virtual component was conceived to provide colleagues from all over the world the opportunity to participate, interact, and learn, regardless of geographical or institutional constraints.

Launched in 2023, IUCS has grown into an inclusive and collaborative forum for clinicians, academic researchers, early-career investigators, industry and non-commercial partners committed to improving outcomes across all stages and subtypes of urologic cancers.

This supplement includes all abstract presentations accepted as of July 14, 2025. Please note that some abstracts may have been revised or withdrawn at the authors’ request.

